# Repurposing of Chronically Used Drugs in Cancer Therapy: A Chance to Grasp

**DOI:** 10.3390/cancers15123199

**Published:** 2023-06-15

**Authors:** Mohamad Ali Hijazi, André Gessner, Nahed El-Najjar

**Affiliations:** 1 Department of Pharmaceutical Sciences, Faculty of Pharmacy, Beirut Arab University, Beirut P.O. Box 11-5020, Lebanon; m.hijazi@bau.edu.lb; 2 Institute of Clinical Microbiology and Hygiene, University Hospital Regensburg, 93053 Regensburg, Germany; andre.gessner@klinik.uni-regensburg.de

**Keywords:** repurposing drugs, oncology, off-targets, anti-hypertensives, anti-diabetic agents

## Abstract

**Simple Summary:**

Cancer mortalities are growing at an alarming pace around the globe, a fact related to challenges, such as side effects, selectivity, and resistance, accompanied by cancer therapy. Continuous development of preventive and therapeutic agents is urgently needed. Drug repurposing is a favored drug discovery strategy resulting in faster, safer, easier, and cheaper repurposed drugs. This review shows the clinical benefits of chronically used anti-diabetic and anti-hypertensive drugs in preventing and treating different human malignancies through a repurposing strategy. The safety, tolerability, effectiveness at low doses, and suitability for long-term use of chronically used medications offer exceptional benefits over other drug classes. Promising clinical evidence exists for anti-diabetic and anti-hypertensive agents for treating different human malignancies. As some have reached Phase IV evaluations, this review offers new insights for clinicians in managing cancer, diabetes, and hypertension.

**Abstract:**

Despite the advancement in drug discovery for cancer therapy, drug repurposing remains an exceptional opportunistic strategy. This approach offers many advantages (faster, safer, and cheaper drugs) typically needed to overcome increased challenges, i.e., side effects, resistance, and costs associated with cancer therapy. However, not all drug classes suit a patient’s condition or long-time use. For that, repurposing chronically used medications is more appealing. This review highlights the importance of repurposing anti-diabetic and anti-hypertensive drugs in the global fight against human malignancies. Extensive searches of all available evidence (up to 30 March 2023) on the anti-cancer activities of anti-diabetic and anti-hypertensive agents are obtained from multiple resources (PubMed, Google Scholar, ClinicalTrials.gov, Drug Bank database, ReDo database, and the National Institutes of Health). Interestingly, more than 92 clinical trials are evaluating the anti-cancer activity of 14 anti-diabetic and anti-hypertensive drugs against more than 15 cancer types. Moreover, some of these agents have reached Phase IV evaluations, suggesting promising official release as anti-cancer medications. This comprehensive review provides current updates on different anti-diabetic and anti-hypertensive classes possessing anti-cancer activities with the available evidence about their mechanism(s) and stage of development and evaluation. Hence, it serves researchers and clinicians interested in anti-cancer drug discovery and cancer management.

## 1. Introduction

Cancer is the most significant health problem burdening the health systems worldwide [1], with more than 27 million expected new cancer cases by 2040 [2]. Cancer mortalities are also growing at an alarming pace around the globe, with nearly 10 million deaths reported in 2020 [3], a number that will reach over 16 million deaths by 2040, according to estimates from the International Agency for Research on Cancer (IARC) [2]. Unfortunately, the dramatically increasing prevalence of risk factors such as smoking, unhealthy diet, physical inactivity, and other environmental factors intensifies this growth [4]. Despite the advancement in cancer biology and the availability of varied therapeutic options [5], the most common malignancies and mortality are increasing, especially in low-income and middle-income countries [6]. Current cancer therapies are subject to multiple challenges related to selectivity, specificity, complexity, adverse effects, and, most importantly, drug resistance [7,8,9,10]. In addition, heterogeneity, a hallmark of all cancers influencing drug responses in nearly all therapeutic modes and all cancer types through different mechanisms, is also a leading cause of drug resistance and, thus, treatment failure [11]. This is unsurprising as tumor heterogeneity is multifactorial, resulting from cancer cells’ differentiation and developmental state, the biology of adjacent normal cells (intrinsic factors), and the tumor microenvironment and genetic mutation (extrinsic factors) [12]. Consequently, the tumors’ highly heterogeneous metabolic profiles represent severe challenges for developing and implementing specific therapeutic strategies [13].

Moreover, long-term use of chemotherapeutic agents, especially those affecting the metabolic pathways and signaling of cancer cells, also affects tumorigenesis, metastasis, drug response, recurrence, drug resistance, and cancer stem cells [14,15,16]. Functional precision or personalized medicine, which follows the dynamic and not the static features to provide information on tumor vulnerabilities and improve patient outcomes, is another strategy in cancer patient therapy [17]. Based on the abovementioned, continuous efforts should be made to rethink therapeutic strategies and develop more effective cancer drugs to overcome these present and future challenges [18].

Drug repurposing represents an exceptional opportunistic strategy in drug discovery [19], where new medical indications are explored for existing, clinically approved off-patent non-cancer drugs [20]. It is like using old weapons for a new battle. The notion of “drug repurposing” includes the search for “off-target” effects or a newly recognized on-target effect of approved drugs to treat other conditions [19,21]. This strategy offers advantages over the classical drug development method in terms of shorter and cheaper drug development processes [21,22]. Discussing the pros and cons of drug repurposing is beyond this review’s scope, and interested readers are referred elsewhere [6,20,21,22,22,23,24,25].

Nevertheless, it is worth noting that several examples of repositioned drugs, including some old drugs such as sildenafil, aspirin, dimethyl fumarate, and others, have marked the history of medicine [22]. In oncology, repurposing, per se, is not a new concept [26]. Successful stories go back to the last century when thalidomide, used initially as a sedative [27], was repositioned and FDA-approved in 1998 for treating multiple myeloma [28]. Arsenic trioxide, a poison, and all-trans retinoic acid, a metabolite of vitamin A, are examples of other chemicals [29] that were FDA-approved in 2000 to treat acute promyelocytic leukemia [30]. Since then, repurposing provided cancer research with new insights into efficiency and efficacy [31]. As a result, today’s literature possesses a surplus of preclinical, observational, and clinical studies and FDA-approved drugs repurposed for cancer therapy. For example, a search on PubMed for “repurposed anti-cancer drugs” results in more than 580 results for the last ten years (2014–2023), with strong increasing trends.

Despite the importance of drug repurposing, irrespective of the types/classes of drugs, some drug classes, such as immunosuppressants [32] and anti-inflammatory [33], could not be used for a long time or are unsuitable for the patient’s condition. Furthermore, antimicrobial drugs might increase the risk of microbial resistance and loss of infection control [34]. In addition, repurposing some classes might be much more complicated and need reformulation or redosing based on the new indication [21]. The potential limitations described above make repurposing of anti-diabetic and anti-hypertensive drugs, chronically used drugs, much more appealing.

## 2. So, Why Is the Repurposing of Anti-Diabetic and Anti-Hypertensive Drugs Exceptional?

Oral anti-diabetic or anti-hypertensive drugs are chronic medications taken daily for an extended period. They are effective at minimal doses, well tolerated, and off-patent, with a well-known safety profile [35]. Therefore, the advantages of chronically used drugs can outweigh the limitations of repurposing other drug classes [36], such as regulatory considerations and patent policies, to state a few [21]. Moreover, the known associations between cancer, diabetes, and cardiovascular diseases, as described hereafter, support repurposing anti-diabetics and anti-hypertensives against cancer.

### 2.1. Relation between Diabetes and Cancer

A strong association exists between diabetes mellitus (especially type 2 diabetes) and carcinogenesis [37]. Both cancer and diabetes are frequently coexisting diseases. Interestingly, many cancers, especially pancreatic, liver, breast, bladder, endometrium, and kidney cancer, have been remarkably linked to diabetes [38]. Thus, an interesting “reverse causality” phenomenon might also exist [39]. Controversial data exist about the relationship between anti-diabetic medications and the incidence and mortality due to cancer [40]. 

Nonetheless, many studies reported the beneficial effects of anti-diabetic drugs in cancer treatment [38]. Anti-diabetic drugs, including sulfonylureas, biguanides, thiazolidinediones, sodium-glucose co-transporter-2 (SGLT2) inhibitors, and dipeptidyl-peptidase IV, have been reported to exert beneficial effects in many cancers’ treatment [41]. The activities of anti-diabetics against cancer are attributed to the metabolic links (i.e., hyperglycemia, hyperinsulinemia, inflammation, oxidative stress, and obesity) between cancer and diabetes [39]. More interestingly, recent reports suggested that anti-diabetic agents can lower cancer incidence directly by impacting the metabolism of cancer cells and indirectly by impacting risk factors of tumors [42]. The most famous example is metformin, the first-line treatment for type 2 diabetes mellitus. Multiple meta-analyses of case-control, cohort studies, and observational and clinical trials have reported its efficacy for cancer prevention and treatment as a single agent or in combination [43]. Metformin significantly reduces the risk of colorectal, breast, pancreatic, prostate, lung [42], and cervical cancer [44]. Other studies report the association of biguanides’ use with a 20–30% reduction in all cancer incidence and cancer-related mortality [45]. In addition, in one cohort study, pioglitazone was associated with decreased risk of renal cancer [46]. Diabetic patients receiving thiazolidinediones reported lower lung cancer rates [47]. Furthermore, recent evidence revealed that SGLT2 inhibitors, a new class of anti-diabetic drugs, were associated with improved overall survival of hepatocellular carcinoma (HCC) patients with pre-existing type 2 diabetes [48]. 

Lastly, the rationale for repurposing anti-diabetic drugs is not surprising considering the link between glucose consumption and cancer, whereby it is well known that tumor cells predominantly use aerobic glycolysis and thus alter metabolic profiles and display elevated glucose uptake [49]. Interestingly, due to increased glucose metabolism in most tumors, positron emission tomography (PET), a non-invasive imaging technique that utilizes ^18^F-fluorodeoxyglucose to provide a functional or metabolic assessment of normal tissue or disease conditions, can and has been used for cancer diagnosis and monitoring of the therapeutic response and to improve further treatment [50,51].

### 2.2. Relation between Cardiovascular Diseases and Cancer

Cardiovascular diseases and cancer possess many similarities and interactions related to analogous risk factors and shared biology, suggesting their medications’ positive effects on each other [52]. For instance, hypoxia, associated with the activation of hypoxia-inducible factor-1 (HIF-1) and nuclear factor κB (NF-κB), plays a critical role in the pathogenesis of hypertension, atherosclerosis, aortic aneurysms, pulmonary arterial hypertension, and heart failure [53,54]. Interestingly, HIF and NF-κB also alter the expression of several genes involved in oncogenic processes, including tumor metabolism, angiogenesis, cancer stem cell specification, cell proliferation, glucose and lipid metabolism, and metastasis [55,56]. Therefore, several HIF inhibitors or activators have been in clinical trials treating patients with advanced/refractory cancers [57,58].

Anti-hypertensive drugs, primarily belonging to the pharmacological categories of angiotensin-converting enzyme inhibitors (ACE), angiotensin II receptors, direct aldosterone antagonists, β-blockers, and calcium channel blockers, can affect the development of malignancy, either directly or indirectly, or both [59]. However, a comprehensive review of data on the role of some anti-hypertensive drug classes in promoting the development of tumors highlights the considerable difficulty of deriving reliable evidence in this setting [60]. While some studies reported the neutral or negative effect of some anti-hypertensive drugs on cancer development [61,62], others provide beneficial effects to either reduction of cancer risks or as promising therapeutics for some human malignancies alone or combined with chemotherapeutic agents [63,64,65]. For instance, diuretics correlate with a higher incidence of liver cancer and lymphoid/hematopoietic tissue cancer development and a lower incidence of prostate and skin cancer [66].

Nevertheless, the involvement of renin-angiotensin and β-adrenergic signaling in blood pressure elevation, cell proliferation, angiogenesis, and tissue invasion highlights the potential importance of inhibiting these pathways for cancer prevention or treatment [67]. In addition, preclinical studies have demonstrated that some anti-hypertensive drugs have a co-adjuvant effect against chemo-resistant cell lines, inhibit cell growth, and increase chemosensitivity in different cancers [63]. For example, a retrospective study shows that using propranolol for over 1000 days reduces the risk of developing various cancer types such as head and neck, esophagus, stomach, colon, and prostate [68]. Other studies show that ACE inhibitors, angiotensin receptor blockers (ARBs), and statins decrease breast cancer recurrence risk [69]. In addition, ARBs and ACE inhibitors, combined with sunitinib, increase the progression-free survival, response rate, and better overall survival and decrease primary treatment refractoriness of patients with renal cell carcinoma (RCC) [70].

This review highlights the exceptional importance of anti-diabetic and anti-hypertensive drugs in the global fight against human malignancies through a repurposing strategy. It provides a comprehensive review of different anti-diabetic and anti-hypertensive classes reported to possess anti-cancer activities with the available evidence about their mechanism(s) and stage of development and evaluation. This study will serve researchers interested in anti-cancer drug discovery. It will also give new insights for clinicians or practitioners in the management of diabetes, hypertension, as well as cancer.

## 3. Repurposing of Anti-Hypertensive and Anti-Diabetic Drugs: Current Update

**Figure 1:**
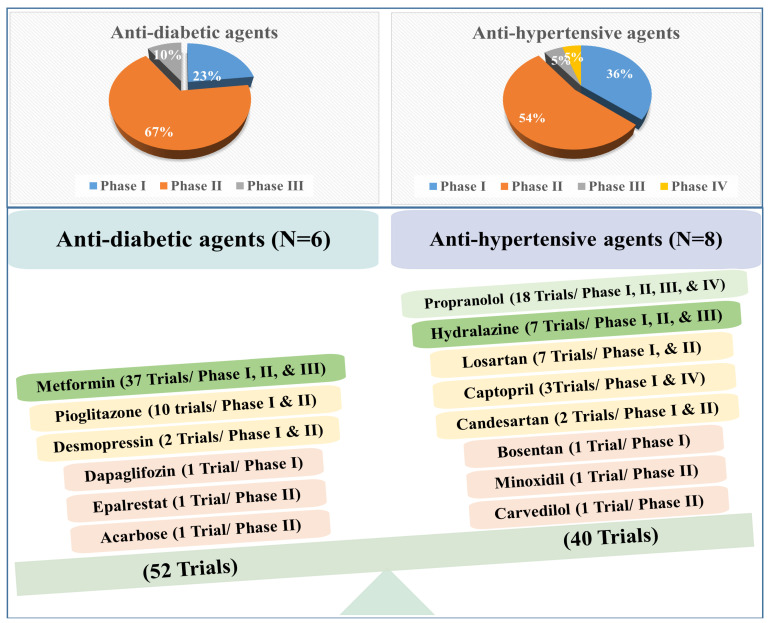
A summary of the percentage of anti-diabetic and anti-hypertensive agents in different clinical trials. The anti-diabetic agents contribute to 52 trials, while the anti-hypertensive agents contribute to 40 clinical trials.

A wealth of data confirms the promising effect of anti-hypertensive and anti-diabetic drugs against cancer. Up to March 2023, 52 and 40 clinical trials evaluated the anti-cancer activity of anti-diabetic and anti-hypertensive drugs, respectively (Figure 1). Interestingly, most of the anti-diabetic and anti-hypertensive agents are in Phase II. For instance, the 52 trials with anti-diabetic agents are in Phase I (23%), Phase II (67%), and Phase III (10%), while the 40 trials of anti-hypertensive agents are in Phase I (36%), Phase II (54%), Phase III (5%), and Phase IV (5%) (Figure 1). 

**Figure 2:**
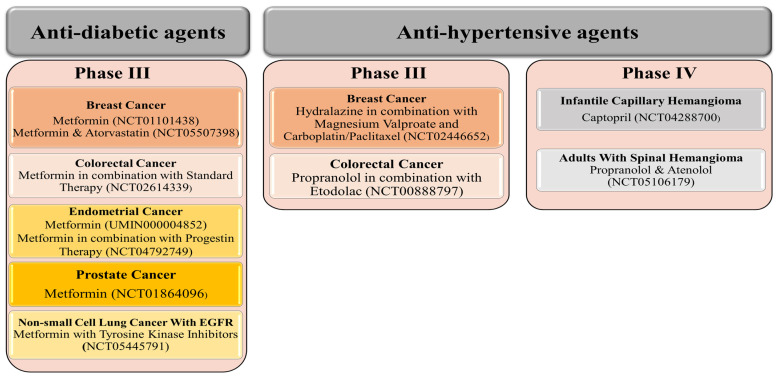
Anti-diabetic and anti-hypertensive agents currently evaluated against different cancer types in clinical Phase III and IV trials. Metformin is the only anti-diabetic agent reaching Phase III against five cancer types. Propranolol and captopril, two anti-hypertensive agents, have reached Phase IV, each against one cancer type.

Although fewer trials are ongoing with anti-hypertensive agents, three anti-hypertensive drugs (propranolol, captopril, and atenolol) have reached Phase IV, each against one benign tumor. For example, captopril is evaluated against infantile capillary hemangioma, while propranolol and atenolol are evaluated in adults with spinal hemangioma (Figure 2). Therefore, we may celebrate the official release of the first anti-hypertensive drug against specific tumor types soon! In addition, other anti-hypertensive agents (hydralazine and propranolol), in combination with other agents, have reached Phase III evaluation targeting breast and colorectal cancer, respectively (Figure 2). 

**Figure 3:**
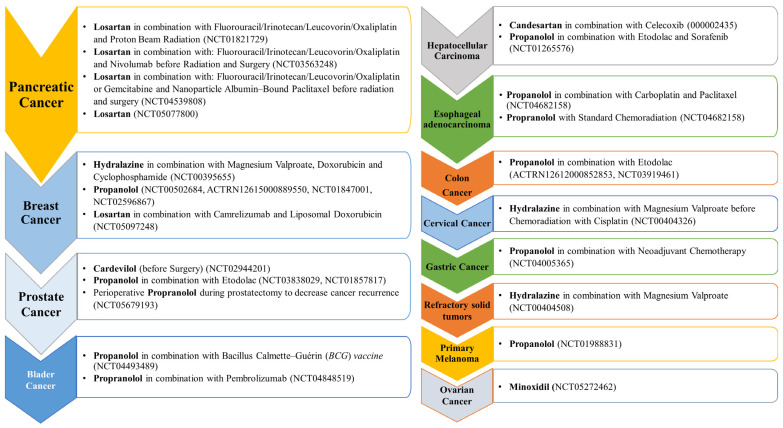
Cancer types listed according to the highest number of Phase II trials using anti-hypertensive agents alone or in combination.

**Figure 4:**
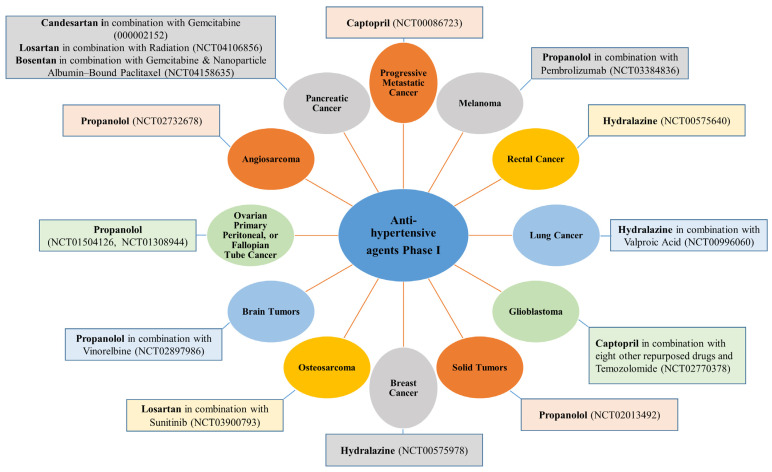
Anti-hypertensive agents currently under evaluation against different cancer types in clinical Phase I trials.

A list of the different anti-hypertensive agents used in Phase I and II against different cancer types are presented, respectively, in Figure 3 and Figure 4. 

**Figure 5:**
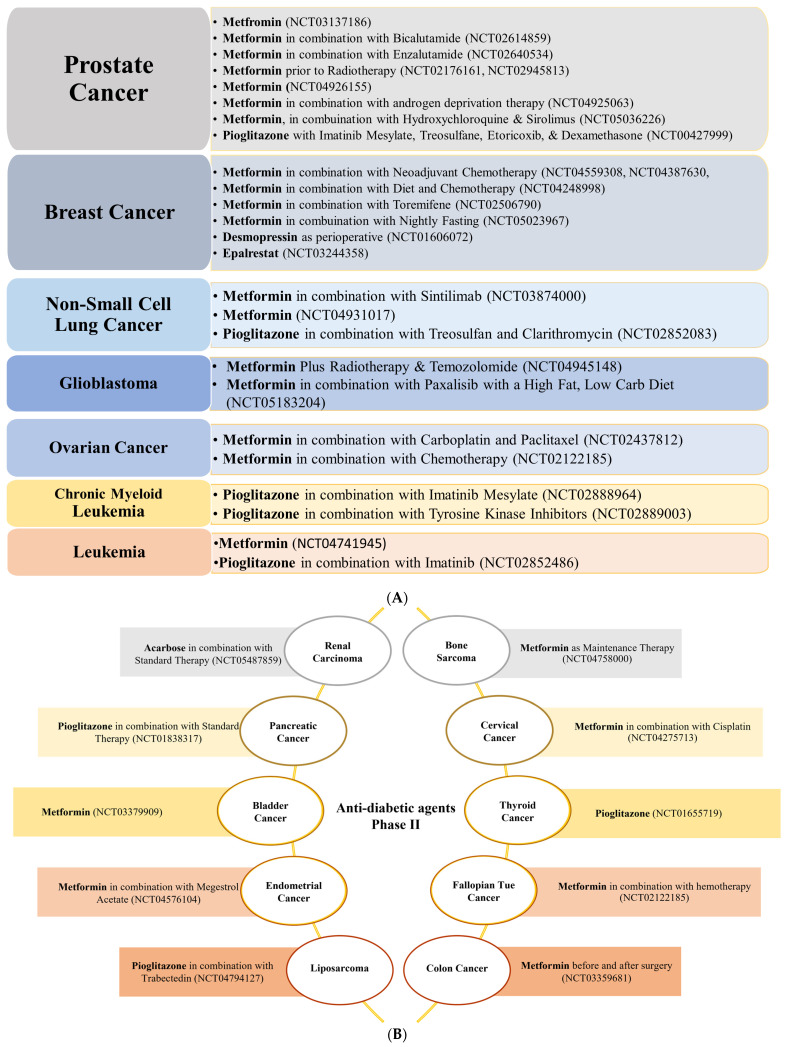
(**A**) A list of cancer types with the highest number of Phase II trials using anti-diabetic agents alone or in combination. (**B**) Cancer types with only one trial currently in Phase II using anti-diabetic agents alone or in combination. Metformin is the most evaluated against six cancer types, followed by pioglitazone (three types) and acarbose (one type).

**Figure 6:**
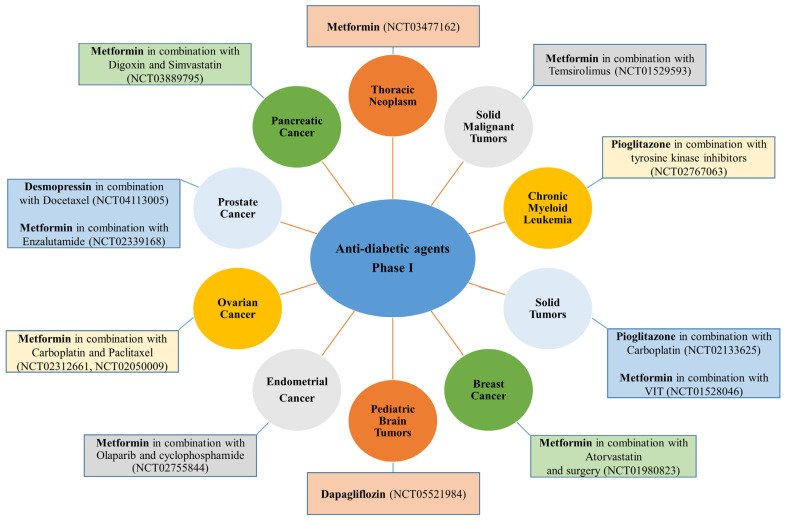
Anti-diabetic agents currently under evaluation against different cancer types in clinical Phase I.

In contrast to anti-hypertensive drugs, none of the anti-diabetic agents has yet reached Phase IV. Nevertheless, metformin is the most popular agent under evaluation, whereby clinical trials.gov presents over 350 trials when searching for metformin and cancer. Filtering the search for active and recruiting studies narrowed the number of trials down to 114 studies. These studies cover trials when metformin is used alone (monotherapy) or with other drugs (combination therapy) to treat cancer. Metformin is the only anti-diabetic agent that has reached Phase III evaluation against five different cancer types either as a single treatment or combined with other therapeutic agents (Figure 2). Other anti-diabetic agents such as pioglitazone and desmopressin currently show high effectiveness against various types of cancers either as a single treatment or combined with other chemotherapeutic agents to decrease cancer cell resistance or improve efficacy (Figure 5A,B and Figure 6). 

The following presents a more detailed overview of the anti-hypertensive and anti-diabetic agents’ usage against different cancer types currently in clinical trials and their reported mechanism of action. 

### 3.1. Colorectal Cancer

#### 3.1.1. Anti-Hypertensive Agents

Propranolol, a non-selective ß-blocker and the most potent anti-hypertensive agent [71], is evaluated in 18 clinical trials as a single treatment or combined with other therapeutic agents (Figure 1). Despite its effectiveness against a wide range of cancers, such as breast, lung, neuroblastoma, angiosarcoma, prostate, and pancreatic, as well as melanoma and leukemia [72,73,74], propranolol has reached Phase II (two trials) (Figure 3) and Phase III (one trial) evaluation against colon and colorectal cancer in combination with etodolac (Figure 2). Mechanistically, propranolol inhibits colorectal cancer by downregulating the expression of p-AKT/p-ERK/p-MEK and simultaneously activating autologous CD8^+^ T cells in vivo [75].

Candesartan and irbesartan (ARBs) decrease tumor vascularization and angiogenesis in colon cancer cell lines [76,77]. In addition, in a patient with metastatic colon cancer, irbesartan inhibits activator protein 1 (AP-1) DNA binding and the JUN gene, resulting in a complete functional radiological resolution of the disease, showing its enormous potential in cancer treatment [78]. ACE inhibitors that block the conversion of angiotensin I to angiotensin II [79], such as enalapril and captopril, are also effective against colon cancer cells in both in vitro and in vivo models [80,81,82]. Enalapril, as a single treatment, reduces insulin-like growth factor 1 receptor (IGF-IR 1) expression [82], while in combination with 5-fluorouracil (5-FU), increases radiosensitivity [80] and shows synergistic activity via the NF-κB/STAT3 pathway [81]. There are no clinical trials with enalapril, but the currently collected data shows its potential as a repurposed drug for cancer. Nifedipine alone suppresses programmed death-ligand1 (PDL-1) expression in colon cancer cell lines [83].

#### 3.1.2. Anti-Diabetic Agents

Metformin, the most commonly used drug for type 2 diabetes, suppresses hepatic gluconeogenesis, improves insulin sensitivity, and enhances peripheral glucose uptake and utilization [84]. Figure 1 shows that metformin is evaluated in 37 clinical trials as a single treatment or combined with standard therapy. However, its activity against colon cancer has reached Phase III trials in combination with standard therapy (Figure 2) and Phase II trials as a single treatment before and after surgery (Figure 4). Mechanistically, metformin leads to cell cycle arrest in different colon cell lines [85]. Unfortunately, no other anti-diabetic agents are currently in phase trials against colon cancer; nevertheless, desmopressin and pioglitazone, two other anti-diabetic agents, showed effectiveness against colon cancer cells via different mechanisms of action. For instance, desmopressin, a selective agonist for vasopressin receptor 2 (AVPR2) [86], was found to inhibit colon cancer cell growth using in vitro and in vivo models [87]. In contrast, pioglitazone, a synthetic peroxisome proliferator-activated receptor (PPAR) ligand that improves glycemic control and insulin sensitivity [88], inhibits, as a single treatment, the metastasis of different colon cancer cells through decreasing cyclooxygenase (COX-2) and cyclin D1 expression [89]. 

### 3.2. Breast Cancer

#### 3.2.1. Anti-Hypertensive Agents

Hydralazine, a vasodilator that relaxes the arteriolar smooth muscles via a poorly understood mechanism [90], is effective against breast cancer and has reached Phase III evaluation in combination with magnesium valproate and carboplatin/paclitaxel (Figure 2). It is also in Phase I and II evaluations as a single treatment or combined with standard therapies, as shown in Figure 3 and Figure 4. Hydralazine increases chemotherapeutic agents’ effectiveness or decreases resistance in breast cancer patients. It can demethylate and re-express silenced *RARβ*, *p21*, and *p16* genes in various breast cancer cells in vivo and in vitro [91,92]. Also, in combination with adriamycin, hydralazine reverses the chemotherapy resistance of breast cancer cells [91]. Propranolol, currently in Phase II (Figure 3), potentiates the anti-angiogenic effect of different chemotherapeutic drugs (i.e., 5-FU and paclitaxel) [93]. Losartan (ARBs), currently in Phase II, in combination with camrelizumab and liposomal doxorubicin (Figure 3), also increases the effectiveness and decreases the resistance of chemotherapeutic agents in different breast cancer cells [94]. In addition, it reduces TNFα, pSTAT3, and IL-6 expression in various breast cancer models in vivo [95]. No other anti-hypertensive agents are currently in trials against breast cancer; however, in vitro data shows the potential effectiveness of other agents against breast cancer. For instance, candesartan, an ARB anti-hypertensive drug, inhibits breast cancer cells T-47D and MCF-7 in vitro [96]. In addition, diazoxide, a potassium channel opener [97], inhibits dual specificity tyrosine-phosphorylation-regulated kinase 1A (DYRK1A), an interleukin-1 receptor-associated kinase 1 (IRAK1), and threonine and tyrosine kinase (TTK) expression in breast cancer cell lines [98]. 

#### 3.2.2. Anti-Diabetic Agents

Metformin stands out for its effectiveness against breast cancer. It is under evaluation in more than seven clinical trials for breast cancer therapy. It has reached Phase III as a single treatment or combined with atorvastatin (Figure 2). Metformin is also in Phase II in combination with chemotherapeutic agents, diet, or fasting, as shown in Figure 5A. Mechanistically, studies revealed that metformin is active against a wide range of breast cancer cells in vitro [43,99,100,101]. For instance, metformin inhibits in vitro cancer stem cells (CSC) via the re-expression of miRNAs and downregulation of CSC-specific genes [102]. It also decreases the phosphorylation of pSTAT3 [103]. Additionally, metformin activates adenosine monophosphate-activated protein kinase (AMPK), decreases the mammalian target of rapamycin (mTOR) and S6 kinase activation, and decreases mRNA translation [104] in breast cancer. Desmopressin is also being evaluated in the Phase II trial as preoperative for breast cancer (Figure 5A). The efficacy of desmopressin against different breast cancer cell lines [105,106,107,108] is through the induction of angiostatin [108] and inhibition of metastasis [109]. Interestingly, a novel analog of desmopressin shows more significant antimetastatic and inhibitory effects against breast cancer cells MCF-7 and MDA-MB-231 [107,108]. Epalrestat, an aldo-keto reductase family 1 member B1 (AKR1B1) inhibitor [110], is in a Phase II study against triple-negative breast cancer (TNBC) (Figure 5A). In opposition to all drugs discussed in this review, it is the only one where no off-site target for its effects against cancer has been discovered. Its anti-proliferative, anti-migratory, and anti-invasive activities originate from its inhibition of AKR1B1. Other in vivo and in vitro studies demonstrated the inhibitory effect of epalrestat against different breast cancer cell lines either as a single treatment or combined with chlorin e6 and photodynamic therapy [111,112]. Pioglitazone, not in clinical trials against breast cancer, induces, in vitro, G_0_/G_1_ arrest via increasing p21 and p27 in MAPK-dependent/partly PPAR-independent manner in MCF7 breast cancer cells [113]. 

### 3.3. Prostate Cancer

#### 3.3.1. Anti-Hypertensive Agents

Carvedilol (a non-selective ß-blocker with alpha1-adrenergic receptor antagonist properties) and propranolol (a non-selective ß-blocker) [71,114] are two anti-hypertensive drugs under evaluation in Phase II trials for prostate cancer (Figure 3). Propranolol is evaluated in combination with etodolac or alone as perioperative, while carvedilol is evaluated in patients with prostate cancer before surgery. A positive impact of other agents has been shown in a retrospective analysis showing that atenolol usage for an extended period significantly lowers prostate cancer incidence by more than 50% [115].

On the other hand, captopril (ACE-I) and candesartan (ARB), not currently in trials, inhibit prostate cancer cells in vivo [116,117] and in vitro [116]. For instance, captopril triggers prostate cancer cells towards self-destruction via increased expression of p53 [117]. Furthermore, candesartan’s tumor growth inhibition in prostate cancer cells was associated with decreased vascular endothelial growth factor (VEGF) expression and tumor angiogenesis inhibition. Interestingly, candesartan did not impair prostate cell viability but inhibited endothelial-barrier disruption by tumor-derived factors [116]. Furthermore, hydralazine leads to the re-expression of silenced genes via demethylation in prostate cancer cells [92,118]. 

#### 3.3.2. Anti-Diabetic Agents

Four anti-diabetic agents are being evaluated in 11 clinical trials for prostate cancer either as a single treatment or combined with other chemotherapeutic agents. The most promising anti-diabetic agent for prostate cancer is metformin, with eight clinical trials, as a single treatment (Phase III, Figure 2) and Phase II (seven trials) either as a single therapy or combined with standard therapies (Figure 5A). Despite the controversial data about the association between metformin use and prostate cancer incidence or survival [115], research has shown that metformin reduces the risk of prostate cancer and improves survival due to its direct anti-cancer mechanisms or secondary effects from the improvement of metabolic syndrome [119,120]). Suggested direct anti-cancer mechanisms include liver kinase B1 (LKB1) and AMPK activation, inhibition of mTOR activity and protein synthesis, induction of apoptosis and autophagy by p53 and p21, and decreased blood insulin [120]. In addition, metformin inhibits prostate cancer cell lines by inducing cell cycle arrest in G_0_/G_1_ via decreased pRb phosphorylation and reduced cyclin D1 and E2F1 protein levels (AMPK independent) [85].

Pioglitazone is in clinical Phase II for prostate cancer in combination with imatinib mesylate, treosulfane, etoricoxib, and dexamethasone (Figure 5A). Population-based case-control study results showed an inverse association between prior pioglitazone usage and prostate cancer [121]. Mechanistically, pioglitazone suppresses levels of TNFα, TGF-β, and the chemokine monocyte chemoattractant protein-1 (MCP-1) [122].

Desmopressin is currently in Phase I in combination with docetaxel (Figure 6). Mechanistically, desmopressin increased the radiosensitivity to docetaxel [86,123,124,125] in different prostate cell lines in vitro and in vivo [86,123,124,125]. Interestingly, the anti-tumor and antimetastatic potential of desmopressin alone has also been shown in prostate cancer via downregulation of the urokinase-type plasminogen activator (uPA) and matrix metalloproteinase (MMP-2 and MMP-9) [125]. 

### 3.4. Pancreatic Cancer

#### 3.4.1. Anti-Hypertensive Agents

More than seven anti-hypertensive drugs show promising anti-cancer activity against pancreatic cancer. Losartan (ARBs class) is in Phase II (four trials) and Phase I (one trial) for pancreatic cancer either as a single treatment or combined with standard chemotherapeutic agents such as 5-FU, irinotecan, leucovorin, oxaliplatin, or before radiation and surgery (Figure 3). It is also effective against different pancreatic cancer cells in vitro [94]. The suggested mechanism for losartan includes reducing stromal collagen and hyaluronan production via a decrease in TGF-β and endothelin-1 (ET-1) expression [94]. Candesartan and bosentan (ET-receptor antagonists, which significantly lower blood pressure in patients with essential hypertension) are in Phase I clinical trials for pancreatic cancer combined with gemcitabine and other therapeutic agents (Figure 4). It has been reported that a potential strategy to selectively enhance tumor perfusion and improve therapeutic agents’ delivery in pancreatic tumors relies on targeting the ET axis [126]. Bosentan exerts antifibrotic and anti-cancer effects in vitro by inhibiting pancreatic stellate cells (PSC) and DSL6A pancreatic cancer cell proliferation and collagen synthesis in PSC [127].

Moreover, recent reports revealed that bosentan enhances gemcitabine’s growth-inhibiting and proapoptotic effects on pancreatic cancer cells by blocking the ET-1/ETAR axis signaling pathway [128]. Enalapril downregulates VEGF expression in pancreatic cancer cells and shows chemopreventive properties in a pancreatic transgenic model [129]. Furthermore, hydralazine shows in vitro promising effects against pancreatic cancer cells by leading to the re-expression of silenced genes via demethylation [92,118,130]. Propranolol alone induces apoptosis, via the intrinsic pathway, in PC-2 cells [116]. Moreover, recent studies confirm that a combination of propranolol and etodolac one week prior to GemNab administration significantly increases overall and progression-free survival in patients with pancreatic cancer [71,131,132,133]. In addition, other anti-hypertensive drugs, nifedipine and fendiline, show promising activity in vitro against different pancreatic cancer cell lines [134,135].

#### 3.4.2. Anti-Diabetic Agents

Only two anti-diabetic agents are in clinical trials for their activity against pancreatic cancer. Pioglitazone, combined with standard therapy, is in Phase II (Figure 5B), while metformin, combined with digoxin and simvastatin, is in Phase I (Figure 6). Pioglitazone alone increases carcinoembryonic antigen (CEA) mRNA expression in various pancreatic cancer cells in vivo and in vitro [136]. Similarly, metformin alone also shows promising inhibitory effects against a wide range of pancreatic cancer cells in vitro and in vivo [85,137,138].

### 3.5. Lung Cancer

#### 3.5.1. Anti-Hypertensive Agents

Hydralazine and valproic acid, in combination, are in a Phase I trial for lung cancer (Figure 4). Both drugs, repositioned as epigenetic agents, exhibit antimetastatic effects in vitro and in vivo against lung cancer cells. Hydralazine and valproic acid treatment induces a growth-inhibitory effect on NIH 3T3-Ras cells, increases gelatinase activity of MMP-2 and MMP-9, and inhibits chemotaxis and cell motility [139]. Emerging data suggest that epigenetic therapies can reprogram the aberrant tumor-associated epigenome and ”tame the beast of resistance”, thereby prolonging survival in non-small cell lung cancer (NSCLC) [140]. 

Other anti-hypertensive drugs, not in clinical trials against lung cancer but showing promising in vitro and/or in vivo effects via different mechanisms of action, are candesartan, captopril, carvedilol, telmisartan, and nifedipine. Candesartan inhibits in vitro protein neddylation in an ATP-competitive manner, thus inhibiting cullin1-Nedd8 and Ubc12-Nedd8 adduction and NAE [96]. On the other hand, losartan inhibits C–C motif chemokine ligand 2 (CCL2) and leads to decreased monocyte recruitment in metastasis [141]. Additional in vitro studies show that telmisartan decreases intercellular adhesion molecule I (CAM1) and MMP-9 expression [142], while nifedipine inhibits the ET-1-induced mitogenic effect of SPC-A lung cancer cells [143]. Moreover, carvedilol inhibits cytochrome P450 1B1 (CYP1B1) in different lung cancer cells in vitro and in vivo [68,144]. In addition, captopril inhibits lung cancer cells LNM35 and induces apoptosis in vitro and in vivo [145].

#### 3.5.2. Anti-Diabetic Agents

Metformin and pioglitazone are the only anti-diabetic agents with promising results against lung cancer. Metformin, combined with tyrosine kinase inhibitors, is in Phase III trials evaluation against NSCLC with EGFR (Figure 2) and Phase II trials as a single treatment or combined with sintilimab (Figure 5A). Metformin’s effects, as a single drug or combined with other therapies, against lung cancer involve a variety of mechanisms that can improve the therapeutic effect and prognosis of lung cancer [146]. Such mechanisms include LKB1-dependent AMPK kinase pathways, AMPK-dependent p53 activation, downregulation of the GRB/IRS-1/PI3K/AKT/mTOR pathway, inhibition of mTORC1 to regulate glucose and amino acid concentration, inhibition of complex I of the mitochondrial respiratory chain, regulation of lung miRNA, modulation of the tumor and its microenvironment (enhanced CD8+ T cell memory), and sensitization of radiotherapeutic agents [140,146,147,148,149].

Similarly, Phase II clinical trials exist for pioglitazone in combination with treosulfan and clarithromycin for NSCLC (Figure 5A). Pioglitazone possesses chemopreventive effects against lung cancer [150] and decreases lung adenoma formation in Benzo (a) pyrene B[a]P in the mouse carcinogenesis model [150]. Furthermore, in an in vivo-induced mouse model of squamous lung carcinoma, treatment with pioglitazone revealed a significant preventive effect [151]. Mechanisms include reduced squamous lesions, increased normal epithelial cells in the airways of exposed mice, and altered EMT gene expression to promote a more epithelial and less mesenchymal phenotype [152]. In addition, it also increases PTEN expression, thus inhibiting the PI3K-Akt pathway [153] and potentiates the effect of gefitinib in lung cancer cell lines [153].

### 3.6. Ovarian, Cervical, and Endometrial Cancers

#### 3.6.1. Anti-Hypertensive Agents

Minoxidil, a direct vasodilator anti-hypertensive agent, is in Phase II trials as a single treatment for ovarian cancer (Figure 3). The mechanism by which minodoxil induces its effect includes altering cancer cells’ metabolic and oxidative state by producing mitochondrial disruption and extensive DNA damage and activating a caspase-3 independent cell death pathway [154]. In addition, two Phase I clinical trials evaluate propranolol for ovarian cancer (Figure 4). In vitro data show that propranolol induces its effect by inducing apoptosis and protective autophagy through the reactive oxygen system (ROS)/JNK signaling pathway [155].

Candesartan and telmisartan, not in clinical trials, inhibit angiogenesis and decrease the expression of VEGF in ovarian cancer cells in vitro and in vivo [76,156]. For instance, based on its ability to demethylate and re-express the silenced *AP* gene, telmisartan increases PPARγ expression and decreases MMP-9 expression in HEY ovarian cancer cells [157]. Hydralazine is being evaluated in Phase II trials for cervical cancer in combination with magnesium valproate before chemoradiation with cisplatin (Figure 3). The combination of hydralazine and valproic acid induces radiosensitization and thus increases the efficacy of cisplatin chemoradiation in cervical cancer [158]. 

Interestingly, preliminary results from a Phase III clinical trial demonstrate a significant advantage in progression-free survival for hydralazine valproic acid (HV) therapy over cisplatin topotecan combination chemotherapy in cervical cancer [159]. Furthermore, epigenetic therapy with HV leads to gene reactivation in primary tumors of cervical cancer patients and protein acetylation (acetylate p53) [160]. Moreover, hydralazine reverts gemcitabine resistance in cervical cancer cell lines by inhibiting G9A histone methyltransferase [161]. In vitro, hydralazine effectively inhibits adenomatous polyposis coli (APC) methylation and promotes APC re-expression, thus inhibiting cell growth in human cervical cancer cell lines [130].

Regarding endometrial cancer, nifedipine and telmisartan are promising therapeutic candidates, inducing their effects by different mechanisms of action. For instance, while nifedipine induces late apoptosis and autophagy via Beclin1 and mTOR pathways in endometrial cancer cell lines [162], telmisartan, on the other hand, induces DNA double-strand breaks and apoptosis [163].

#### 3.6.2. Anti-Diabetic Agents

Metformin, with ten clinical trials, is the only tested anti-diabetic agent against ovarian, cervical, and endometrial cancers. Fascinatingly, metformin is in Phase III trials either as a single treatment or combined with progestin for endometrial cancer (Figure 2). Moreover, five Phase II trials also exist for metformin in combination with chemotherapeutic agents for ovarian cancers (two trials, Figure 5A), endometrial, fallopian, and cervical cancer (one trial each, Figure 5B). Three additional Phase I trials evaluate metformin against ovarian and endometrial cancer (Figure 6). Mechanistically, metformin decreases the proliferation, migration, and invasion of patient-derived ovarian cancer cells [164,165] by decreasing sphingosine kinase-1 (SPHK) expression and reducing serum sphingosine-1 phosphate (S1P) levels. In addition, it causes apoptosis in ovarian and gastric cancer via ROS-mediated apoptosis signal-regulating kinase 1 (ASK1) activation and JNK phosphorylation and Noxa expression leading to loss of mitochondrial potential and hence caspase-3 cleavage [165,166]. On the other hand, pioglitazone treatment increases apoptosis and necrosis and decreases VEGF expression and microvessel density (MVD) in solid OVCAR-3 tumors. Combined with clofibric acid, pioglitazone produces a potent anti-tumor effect on ovarian cancer by reducing AP-1 expression [167].

### 3.7. Cancers of the Brain and Spinal Cord, Neuroblastoma, Osteosarcoma, and Head and Neck Squamous Cell Carcinoma 

#### 3.7.1. Anti-Hypertensive Agents

The most remarkable and exciting effects are related to captopril, propranolol, and atenolol, which have reached Phase IV clinical evaluation and could obtain FDA approval at any time. For example, captopril is in the final Phase (Phase IV) for infantile capillary hemangioma, while propranolol and atenolol are for adults with spinal hemangioma (Figure 2). Moreover, captopril, combined with eight other drugs, is also in Phase I against glioblastoma, while propranolol, combined with vinorelbine, is in Phase I against brain cancer (Figure 4). 

Mechanistically, a study by Sulzberger et al. shows that treating infantile capillary hemangioma with captopril confirms the renin-angiotensin system’s significant contribution to the disease’s biology. Captopril decreased the mean levels of angiotensin II without affecting the mean levels of ACE [168].

Moreover, inhibition of MMP-2 expression plays a significant role in the anti-invasive effect of captopril alone and when combined with temozolomide against gliosarcoma in vitro and in vivo [169]. Additionally, propranolol induces apoptosis via the p53 pathway in neuroblastoma cells [170] and synergizes chemotherapeutic effects in HNSCC [171] and in neuroblastoma [172]. No clinical trials are currently conducted with nifedipine, yet it synergies cisplatin’s effect in glioblastoma cells [173]. Other anti-hypertensive agents such as carvedilol and losartan also show interesting inhibitory effects. For example, carvedilol potentiates vincristine’s effects in neuroblastoma cells and TH-MYCN transgenic mice [172], while losartan inhibits glioma cells C6 in Wistar rats [174]. Losartan is also in Phase I, combined with sunitinib, for treating osteosarcoma (Figure 4).

#### 3.7.2. Anti-Diabetic Agents

Dapagliflozin, an SGLT2 inhibitor [175], is now in a Phase I clinical trial against pediatric brain tumors (Figure 6). In addition, three Phase II trials are registered for metformin as treatment or maintenance therapy for glioblastoma and bone sarcoma (Figure 5A,B). A wealth of clinical and preclinical studies support the potential of metformin against glioblastoma due to its ability to strongly support the standard care strategy toward improving overall and progression-free survival [176]. For instance, metformin hyperactivates the AMPK pathway and plays a role in apoptosis induction, cell proliferation reduction, metastasis inhibition, and chemo-radio-sensitizer behavior against glioblastoma multiforme [177]. However, a more profound knowledge of the anti-angiogenic effect of metformin in glioblastoma is needed. 

The most remarkable effect of pioglitazone includes the induction of ferroptotic cell death HNC cells [178] and its depletion of stem cells, thus leading to its antimetastatic effect [89,136]. Combined with sulfasalazine, it inhibits CDGSH iron sulfur domain 2 (CISD2), thus increasing lipid ROS and ferrous iron levels. Furthermore, it sensitizes sulfasalazine-resistant cells to ferroptotic cell death against a wide range of HNC cells in vivo and in vitro [178]. Another anti-diabetic agent, desmopressin, inhibits osteosarcoma cells MG-63 in nude mice and U2-OS cells in vitro [179].

### 3.8. Liver and Kidney Cancer

#### 3.8.1. Anti-Hypertensive Agents

Despite the absence of clinical trials evaluating their effectiveness against liver or kidney cancer, four anti-hypertensive drugs (candesartan, irbesartan, telmisartan, and captopril) are promising candidates based on in vitro and in vivo studies [96,180,181]. For instance, telmisartan and irbesartan inhibit liver cancer cells by reducing pErbB3 expression [181] and p38/MAPK phosphorylation leading to a reduction in VCAM-1 expression [180]. In addition, telmisartan induces apoptosis in kidney or renal cancer cell lines via increased caspase-3 and Bax expression and decreased Bcl-2 expression (PI3/AKT pathway) [163,182,183]. Captopril, in turn, inhibits kidney cancer cells SN12K-1 in vivo and in vitro [184]. 

#### 3.8.2. Anti-Diabetic Agents

Acarbose, a complex oligosaccharide that acts as a competitive, reversible inhibitor of pancreatic alpha-amylase and membrane-bound intestinal alpha-glucoside hydrolase [185], is in Phase II clinical trial in combination with standard chemotherapy for treating renal sarcoma (Figure 5B). Although its mechanism for metastasis inhibition is unclear, studies found that acarbose slowed kidney cancer growth and promoted protective immune responses [186]. Moreover, combined with either immunotherapy or targeted therapy, acarbose improved renal cancer treatment outcomes and reduced lung metastases [186]. Again, pioglitazone has a chemoprotective effect on HCC [187]. This effect is due to pioglitazone’s ability to decrease fibrosis to HCC progression in DEN rat models through increased adiponectin production, reduced MAPK activation, and increased AMPK activation [187]. Furthermore, pioglitazone induces apoptosis and cell cycle arrest in G_0_/G_1_ by decreasing expression of RAGE, NF-κB, HMGB1, p38MAPK, Ki-67, MMP-2, and cyclin D1 in human HCC tissues [188]. In addition, metformin affects the promotion of cell growth under low glucose conditions in renal cell carcinoma [189] and inhibits Rag GTPases and mTORC1 signaling (AMPK independent) in stem cells [190] and kidney cancer [191]. 

Moreover, epalrestat also inhibits liver [192] and kidney [193] cancer cell lines. Different mechanisms are reported for the anti-cancer activity of epalrestat as a single treatment or combined with chemotherapy and targeted therapeutics in experimental models of liver cancers [194]. These include inhibition of NF-κB signaling through inhibition of AKR1B1 [111,195], inhibition of proliferation, migration, invasion, and metastasis [111,112,196], induction of G_0_/G_1_ cell cycle arrest [195] via p27/p-Rb pathway (reduced expression of p-Rb, cyclin D1, and E) [197], and induction of apoptosis and autophagy via mTOR inhibition [195,198]. In addition to inhibition of AKR1B1 and/or AKR1B10 and blockade of the epithelial-mesenchymal transition, epalrestat synergizes daunorubicin and idarubicin’s effects [193] and enhances sorafenib’s inhibitory effects in vivo and in vitro [192,198].

### 3.9. Gastric and Esophageal Cancer 

#### 3.9.1. Anti-Hypertensive Agents

Propranolol is in Phase II trials combined with neoadjuvant chemotherapy for gastric cancer (Figure 3). The mechanism by which propranolol inhibits tumor growth and proliferation of gastric cancer cells is by inducing G_1_-phase cell cycle arrest and apoptosis [199]. It also decreases the expression of phosphorylated CREB-ATF and MEK-ERK pathways, suppresses the expression of MMP-2, MMP-9, and VEGF, and inhibits gastric cancer cell migration [199,200]. Furthermore, propranolol, by inhibiting the expression of NF-κB, EGFR, COX, VEGF, and other factors, improves the radiotherapy effects of oxaliplatin and tigio against gastric cancer [201].

Although not yet in clinical trials, other anti-hypertensive drugs such as candesartan and captopril inhibit different gastric cancer cell lines in vitro [96] and in vivo [202]. Captopril also inhibits gastric cell invasion by reducing the activity of MMP-2 and MMP-9 [202]. On the other hand, telmisartan is effective against a wide range of esophageal [203,204] and cholangiocarcinoma cancer cell lines in vitro and in vivo [205]. Telmisartan’s effects are due to its ability to reduce pErbB3 and thrombospondin-1 expression [204] and to induce S-phase arrest via reduction of cyclin A2, E, and D1 and CDK2 expression, thus leading to mTOR inhibition [181,203,204,205,206].

#### 3.9.2. Anti-Diabetic Agents

No clinical trials are available for any anti-diabetic agent for gastric or esophageal cancer. However, in recent years, several observational studies have shown that metformin reduces the risk of gastric cancer [207,208]. In addition, studies suggested that metformin alone decreased tumor volume, while combined with cisplatin, rapamycin, or both increased the effect of each drug alone and inhibited the peritoneal dissemination of gastric cancer [209]. Others reported that metformin induces pyroptosis in esophageal squamous cell carcinoma in mice by elevating cleaved GSDMD (miR-497/PELP1 axis) [210]. In addition, metformin induces apoptosis and suppresses the mTOR/AKT pathway in AMPK-dependent pathway [165,166].

### 3.10. Skin Cancer

#### 3.10.1. Anti-Hypertensive Agents

Telmisartan influences the metabolism of melanoma cells and increases glucose uptake but not glycolysis, which leads to mitochondrial fission, ROS generation, and apoptosis induction in skin cancer [211]. Telmisartan also enhances vemurafenib’s effects against skin cancer cells [211]. Propranolol, as a single treatment or combined with other anti-cancer agents, affects the skin cancer cells via multiple mechanisms [212,213,214]. For instance, propranolol increases p53 and decreases Akt3, PiK3R5, and HIF1a expression [215]. It also induces apoptosis via increasing Bax/Bcl-2 ratio [216]. Moreover, carvedilol inhibits the EGF-mediated cell transformation of JB6 P+ cells by inhibiting AP-1 [68].

#### 3.10.2. Anti-Diabetic Agents

In vitro and in vivo studies confirm the effectiveness of metformin against skin cancer via different mechanisms. For instance, metformin arrests melanoma cells in the G_0_/G_1_ phase [217] and attenuates melanoma growth and metastasis by inhibiting the expression of TRB3 (tribbles pseudokinase 3) in vivo [218]. In addition, it can also influence melanoma cell death and proliferation and the tumor microenvironment via the activation of AMPK. Furthermore, metformin also decreases cell growth and invasion of melanoma through modulation of miR-192-5p/EFEMP1 and miR-584 3p/SCAMP3 axes in a wide range of melanoma cell lines [219].

### 3.11. Blood Cancer

#### 3.11.1. Anti-Hypertensive Agents

Preliminary results of a Phase II study suggest that combining hydralazine and valproate is a promising non-toxic and effective therapy for myelodysplastic syndrome (MDS) [160]. Furthermore, exciting in vitro findings reported the ability of hydralazine to inhibit T-cell leukemia cells Jurkat [220]. Suggested mechanisms include the inhibition of DNA methylation in T cells [220], the mRNA expression of DNA methyltransferases (DNMT) 1 and 3A in Jurkat cells [118,220], and the EGFR pathway [118,220]. On the other hand, telmisartan induces autophagy and caspase-dependent and independent cell death in different adult T-cell leukemia cells [221]. Enalapril is also active against blood acute promyelocytic leukemia (APL) cells by reducing STAT5 expression [222]. Other findings suggest that captopril has a significant benefit in overcoming pathological changes associated with myelofibrosis [223]. Suggested mechanisms are the normalization of bone marrow cellularity, reducing reticulin fibers, splenomegaly and megakaryocytosis, and inhibiting collagen expression [223]. 

#### 3.11.2. Anti-Diabetic Agents

Pioglitazone and metformin are repurposed for leukemia. Three Phase II trials and one Phase I evaluate pioglitazone’s efficacy when combined with other agents (imatinib and tyrosine kinase inhibitor (TKI)) against leukemia or chronic myeloid leukemia (Figure 5A). Mechanistically, pioglitazone arrests Jurkat l, T-cell leukemia cells, in the G_2_/M phase, by increasing p27 and decreasing cyclin D1 expression [224,225,226]. In addition, pioglitazone depletes stem cells via decreased STAT5, HIF2α, and carboxy-terminal domain 2 (CITED2) expression in chronic myeloid leukemia (CML) [227] and potentiates the effect of imatinib [227]. One Phase II trial also investigates metformin’s efficacy against leukemia (Figure 5A). In addition, a large body of evidence supports the effectiveness of metformin against hematological malignancies, including leukemia, lymphoma, and multiple myeloma, expected to be due to its pleiotropic effects on multiple targets [228]. Reported data suggest that metformin induces necrosis and apoptosis in myeloma cells [229]. In addition, metformin inhibits cell growth by arresting the cells in G_0_/G_1_ or G_2_/M phase, activating AMPK, inducing caspase-dependent apoptosis, and inhibiting the expression of Mcl-1, IGF-1R, PI3K, pAKT, and pmTOR proteins [228,229].

## 4. Conclusions and Future Prospects

Traditional drug development procedures have come at a high cost of anti-cancer drugs, significantly increasing the global burden on health economies [5]. Accordingly, “financial toxicity”, which describes monetary and health implications related to the financial burden of receiving care, became a familiar term in assessing cancer drugs and is gaining consideration in successful cancer management [230]. In addition, the skyrocketed prices of anti-cancer medications harm the patients’ mental health (causing distress, stress, hardship, and emotional strain) and their families [24]. Furthermore, a substantial portion of cancer patients is not accessing or receiving adequate care mainly because of disparities in access to cancer care and high financial cost [24]. Collectively, these challenges emphasized the need to improve success rates, shorten processing time, and cut costs in cancer drug development via alternative ways [25]. Therefore, repurposing has become a hot topic, especially in cancer therapy. Many studies evaluate and report possible off-target activities of different drug classes for therapeutic applications, including chemotherapy. However, despite the importance of drug repurposing, it is challenging, and clinical benefits cannot be easily achieved. In addition, barriers such as regulatory considerations, patent policies, the new business model, and organizational and industrial obstacles still exist [21].

Nevertheless, repurposing anti-diabetic and anti-hypertensive drugs to reduce the incidence of some cancer types and assist in preventing or treating human malignancies is unique and exceptional [39], as previously discussed. Several lines of evidence from in vitro, in vivo, and clinical trials support the importance of anti-diabetic and anti-hypertensive agents in the fight against cancer. This is not surprising considering that diabetes and hypertension (both are common comorbidities) are also closely interlinked to cancer due to similar risk factors [231]. The link between these ailments offers different opportunities by having different and similar targets that can be exploitable for treating cancer. 

Nonetheless, an exciting conclusion of this review shows that among the eight anti-hypertensive agents (propranolol, hydralazine, losartan, captopril, candesartan, bosentan, minoxidil, and carvedilol) and the six anti-diabetic agents (metformin, pioglitazone, desmopressin, dapagliflozin, epalrestat, and acarbose), only epalrestat initiates its effects in breast, cervical, oral, liver, and kidney cancers by inhibiting AKR1B1/AKR1B10, the same target in diabetes and cancer [111,195]. Another exciting anti-diabetic drug is metformin, which inhibits different cancers by modulating similar on-targets (diabetes and cancer) and off-targets (only in cancer). For instance, metformin induces cell cycle arrest (colon [85], skin [217], and myeloma [228,229]), induces apoptosis (prostate [120], ovarian [164,165], glioblastoma [177], and myeloma [228,229]), induces pyroptosis (esophageal squamous cell carcinoma [210]), induces autophagy (prostate [120]), decreases metastasis (colon [102,164], ovarian [164,165], glioblastoma [177], and skin [217]), and inhibits CSC (breast [102,137] and kidney [191]). In addition, the pathways modulated repeatedly by metformin are the STAT pathway (breast [103,138]), and the mTOR pathway (breast [104,166], prostate [120], kidney [191], and myeloma [228,229]). Nevertheless, the striking effect of metformin is inducing its effect by activation of AMPK (AMPK dependent), the same on-target in diabetes, in the lung [140,146,147,148,149], glioblastoma [177], skin [217], and myeloma [229]. Therefore, it is no wonder that metformin is in 37 trials due to its pleiotropic effects. 

Other anti-hypertensive and anti-diabetic agents induce their effects by targeting different off-targets in different cancer types, evidenced by the current reported data. For example, pioglitazone, the second most evaluated anti-diabetic agent in trials (10 trials), shows only different off-targets in cancer. For instance, pioglitazone induces cell cycle arrest (breast [113], leukemia [224,225,226], and liver [188]), induces apoptosis (liver [188] and ovarian [167]), induces necrosis (ovarian [167]), induces ferroptosis (head and neck [178]), inhibits angiogenesis (ovarian [167]), and inhibits metastasis (colon [89], head and neck [178]). In addition, pioglitazone also plays significant anti-inflammatory roles in the colon [89] and prostate cancer [122] by decreasing COX, TNF, and MCP-1. 

Similar diversity of effects is also observed with the anti-hypertensive drugs, yet hydralazine is a remarkable anti-hypertensive drug (seven trials) that induces its effects by inducing epigenetic alterations (DNA demethylation) in different cancers such as breast [91,92], pancreas [92,118], and cervical [130]. Hydralazine’s effects range from DNA demethylation of different targets to inhibition of DNMT and G9A histone methyltransferase. Conversely, it is worth mentioning that further studies will be needed to clarify the benefit-to-risk ratio, considering hydralazine’s effect on DNA methylation that might lead to autoimmunity and lupus-like disease [220,232]. 

Moreover, the controversial data regarding the risks of developing cancer associated with anti-hypertensive and anti-diabetic drugs merits further analysis. For instance, despite their beneficial effect, metformin and thiazolidinediones could associate with an increased risk of bladder cancer [42]. Furthermore, whether SGLT-2 inhibitors have anti-cancer potential or are potentially harmful is still unanswered [42]. Although randomized clinical trials are ethically unfeasible, observational studies usually present biases that limit their reliability. For this reason, “big data” need to be collected more in-depth to produce evidence-based recommendations [233]. All in all, recommendations to interrupt successful anti-hypertensive therapies to avoid a generic risk of cancer do not appear to be easily justified [233].

Notably, this review reports data based on clinical and preclinical studies and has some limitations, as discussed hereafter. For instance, published preclinical data do not necessarily ensure drug approval and, thus, the success of therapeutics in cancer [234]. Moreover, only reporting the author’s aims and conclusions of clinical trials may not always be sufficient to make the proper clinical conclusion [235]. For instance, although multiple observational studies reviewed in numerous systematic reviews have shown that anti-diabetic treatment may reduce the risk of cancer and improve the efficacy of cancer treatment in diabetic patients [43,236,237,238,239], clinical studies that attempted to show similar protective effects in non-diabetic cancer patients revealed controversial results and have been disappointing [240]. The potential optimal dose, schedule, and duration of treatment and the heterogeneity of histological subtypes and genotypes among cancer patients could explain these differences in clinical benefits between diabetic and non-diabetic patients. Similar conclusions were also reported for anti-hypertensive drugs in cancer patients [67].

Therefore, it would be ideal to critically appraise the quality, internal and external validity, homogeneity, study design, consistency of results, analysis, clinical and statistical significance, and any bias or conflicts of each clinical trial to provide sufficient details and avoid significant gaps that can lead to misleading results [241,242]. Furthermore, clinical trials evaluating the immunoregulatory effects of anti-diabetic and anti-hypertensive drugs on cancer are needed. In addition, assessing data generated after the long-term use of approved drugs is essential to evaluate their success. 

Nevertheless, despite the abovementioned limitations, the data presented in this review show how valuable it is to repurpose drugs that can be effective against different cancer types via multiple mechanisms.

Finally, in this review, a summary of the current information regarding the clinical trials and other available studies is also presented and discussed. In addition, the benefits of exploiting anti-diabetic and anti-hypertensive agents, chronically used drugs, for treating different cancers by modulating on and off-site targets are highlighted. Unfortunately, despite the wealth of available data about the importance of drug repurposing, current therapy relies on expensive drugs. Therefore, cancer patients need a fast availability of cheap, effective, and non-toxic drugs. Hopefully, with the many promising trials with safe anti-hypertensive and anti-diabetic drugs, all cancer patients will be treated without disparities, and the financial burden will no longer be a significant issue.
